# Effect of Environmental Disturbance on the Population of Sandflies and *Leishmania* Transmission in an Endemic Area of Venezuela

**DOI:** 10.1155/2014/280629

**Published:** 2014-04-07

**Authors:** Elsa Nieves, Luzmary Oraá, Yorfer Rondón, Mireya Sánchez, Yetsenia Sánchez, Masyelly Rojas, Maritza Rondón, Maria Rujano, Nestor González, Dalmiro Cazorla

**Affiliations:** ^1^LAPEX-Laboratorio de Parasitología Experimental, Departamento de Biología, Facultad de Ciencias, Universidad de Los Andes, Mérida 5101, Venezuela; ^2^LEPAMET-Laboratorio de Entomología, Parasitología y Medicina Tropical, Universidad Nacional Experimental Francisco de Miranda, Falcón 4101, Venezuela

## Abstract

The exploitation of new wilderness areas with crops is increasing and traditional crop substitution has been modified by new more productive crops. The results show the anthropogenic disturbance effect on the sandflies population and *Leishmania* transmission in endemic areas of Venezuela. Three agroecosystems with variable degrees of ecological disturbance, forest (conserved), cacao (fragmented), and orangery (disturbed), were selected. Four methods to sandfly capture were used; the specimens were identified and infected with *Leishmania*. Diversity, population structure, ANOVA, Tukey test, and simple correlation analysis were carried out. Shannon traps were able to capture 94.7% of the total sandflies, while CDC light traps, Sticky traps, and direct suction just captured 2.2%, 1.2%, and 0.9%, respectively. The results showed the effect of ecological disturbance degree on the composition of sandflies and population structure, revealing a dominance level increased but decreased on the diversity and richness of sandflies species in the greatest ecological disturbance area in relation to areas with less organic disturbance. Environments more disturbed cause adaptability of certain species such as *Lutzomyia gomezi* and *Lutzomyia walkeri*. These changes on the composition of sandflies population and structure emerging species could cause increasing of leishmaniasis transmission.

## 1. Introduction

The distribution of sandflies correlated with the appearance of cases of leishmaniasis in endemic regions, especially in forested areas. However, with human intervention and the disappearance of their natural habitat, some species appear to have adapted to degraded habitats, contributing to expansion of their spatial distribution and the spread of leishmaniasis [1–3].

The main factors involved in the transmission of tegumentary leishmaniasis are related to deforestation, urbanization, the presence of domestic animals, and the development of agriculture, particularly the cultivation of cocoa, banana, and coffee [4]. Anthropogenic factors tend to alter the composition and behavior of populations of sandflies. While some species of sandflies have disappeared, others have become more abundant and have adapted to synanthropic environments by changing their behavior [5–10].

The exploitation of wilderness areas for cultivation is increasing. In particular, this expansion has replaced traditional crops with crops that are more productive, which has led to changes in sandflies populations related to altered patterns of dispersal and spatial distribution of these species in new areas [10–14], because these changes may involve a greater risk of transmission [3, 15]. Thus, an understanding between habitat variation and sandflies populations is essential, and to examine whether these changes can increase the risk of transmission of* Leishmania*, we studied populations of sandflies in a conserved area and two distinct agroecosystems.

## 2. Materials and Methods

### 2.1. Study Area

The agroecosystems located in the Parroquia Caño El Tigre, Zea Municipality, Merida, Venezuela, were studied. These regions have an average elevation of 300–400 meters above sea level, covering an area of 135 km^2^, which includes 9,595 inhabitants, a tropical rainforest climate, and temperatures that range between 25 and 30°C. The main economic activities of the region are agriculture and cattle.

### 2.2. Determination of Environmental and Anthropogenic Variables

According to methods previously described in the literature, indicators associated with ecoepidemiological levels were recorded using a data sheet that identified the environmental and anthropogenic variables related to the presence of sandflies. These variables included the climatic conditions (elevation, temperature, and relative humidity), the presence of natural or anthropogenic water bodies, dominant vegetation stratum, crops and animals present, and the level of human interference (e.g., logging, burning, and use of fertilizer).

### 2.3. Degrees of Disturbance

**Figure 1:**
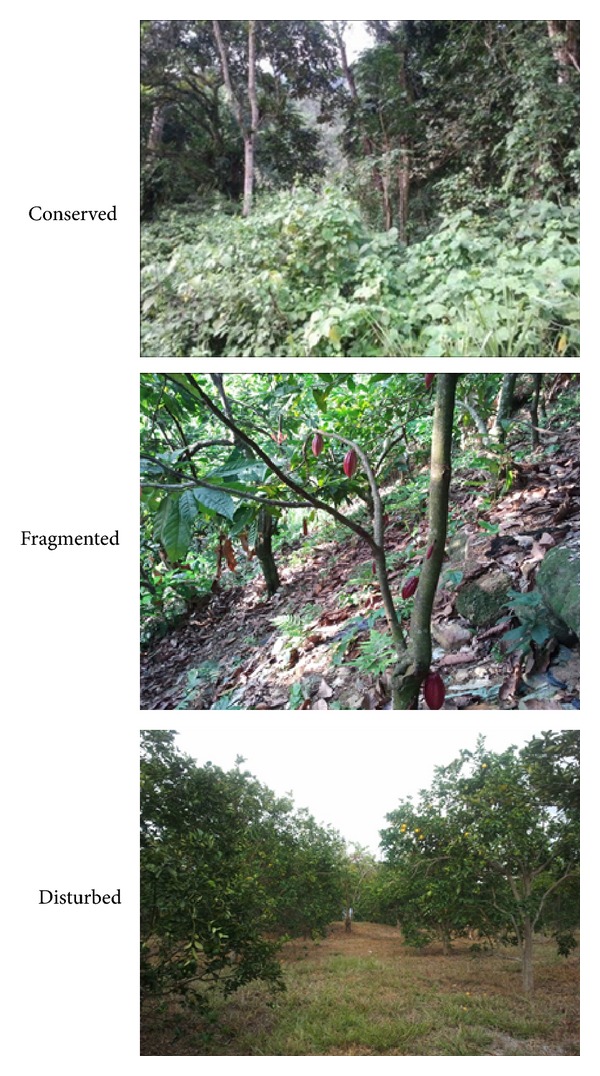
Different degrees of ecological disturbance of the agroecosystems.

The agroecosystems were characterised according to the degree of human modification [3]. The aspects observed concern the vegetation and the presence of both dwellings and animal shelters. Three agroecosystems were selected with varying degrees of ecological disturbance: (1) a conserved area, predominantly forest, characterized by abundant primary vegetation; (2) a fragmented area in which primary vegetation was partially replaced by cocoa crops without management; and (3) a disturbed area with complete replacement of primary vegetation, resulting from the degradation caused by human activity related to citrus cultivation, specifically oranges (Figure 1). The distance between the agroecosystems is approximately 10 km.

### 2.4. Capture of Sandflies

Captures of adult sandflies specimens were performed for 12-month period, from January 2012 to January 2013 at three agrosystems. The captures were conducted at the peridomicile areas, using one Shannon traps, three CDC traps, six Sticky traps, and direct suction with an oral grabber. Sampling was conducted after sunset, when sandflies are most active, between 18:30 h and 20:00 h; with minimum of one capture by months each collection agrosystem. Shannon traps were conducted in peridomicile areas with three collectors, the CDC light traps were placed in proximity of houses (poultry houses, breeding pigs, tree, etc.), and Sticky strips (white paper sheets 21.6 × 27.9 cm coated with castor oil) were placed indoors or outdoors in proximity of houses. The traps were distributed over 1 ha of the agrosystems and arranged in transect with at least 20 m of distance between each trap.

### 2.5. Determination of Natural Infection and Sandflies Identification

To determine the presence of* Leishmania *promastigotes [16], the digestive system was extracted via the dissection of live females and examined using phase contrast microscopy at 400x magnification. We then performed rapid identification of fresh sandflies individuals, and body or representative segments were subsequently cleared in Nesbitt solution for 24 hours and were prepared and mounted on slides using Berlese's medium to identify females for corroboration of the species by comparative external and internal morphology [17].

### 2.6. Analysis

The methods used were based on community structure, proportional abundance, dominance index, and Margalef's index which was used to calculate biodiversity [18]. An analysis of the different captures among and agrosystems was conducted using a cluster analysis which was performed using PCORD.5 software (License belonging to ICAE). The comparison for the different agrosystems was conducted using analysis of variance (ANOVA) which was performed with a level of significance of 0.005, Tukey's test. To investigate the possible association between species distribution and ecosystems a simple correspondence analysis was carried out using the IBM SPSS statistical software package, which is publicly available for download at http://ibm-spss-statistics.softonic.com.

## 3. Results

The ecoepidemiological characteristics and the degree of disturbance of the 3 agroecosystems are summarized in Table 1. The environmental characterisation demonstrated that the studied areas presented different degrees of anthropogenic modification. The forest was more preserved and the orangery was more modified.

The Shannon traps, CDC light traps, Sticky traps, and direct suction captured 94.7%, 2.2%, 1.2%, and 0.9% of the sandflies, respectively.* L. gomezi *was the most abundant species in the area, present in all environments studied. According to the abundance values of sandfly specimens collected,* L. gomezi, L. ovallesi*,* L. walkeri, L. trinidadensis, *and* L. panamensis *were the main species identified in the 3 agroecosystems. These species were found at different abundance levels, although* L. panamensis *was only detected in the conserved forest (Table 2).

**Figure 2:**
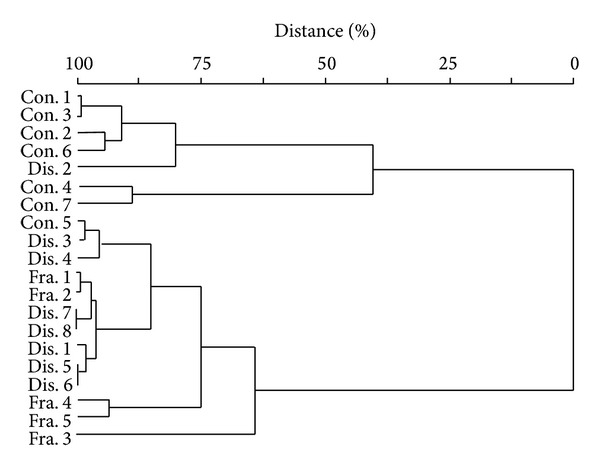
Cluster analysis of the capture in the agroecosystems.

Cluster analysis was performed to assess the segmentation of each capture, and we identified 2 groups of homogeneous captures with 46% similarity. These groups corresponded to captures in conserved and fragmented environments (Figure 2).

The ANOVA results showed significant differences between the populations of sandflies identified in each agroecosystem (one-way ANOVA,* F* = 551, df = 16, *P* = 0.000). To further evaluate these differences, a* post hoc *Tukey's test was performed for paired agroecosystems, specifically between forest and cocoa agroecosystems and cocoa and orange agroecosystems (Table 3).

**Figure 3:**
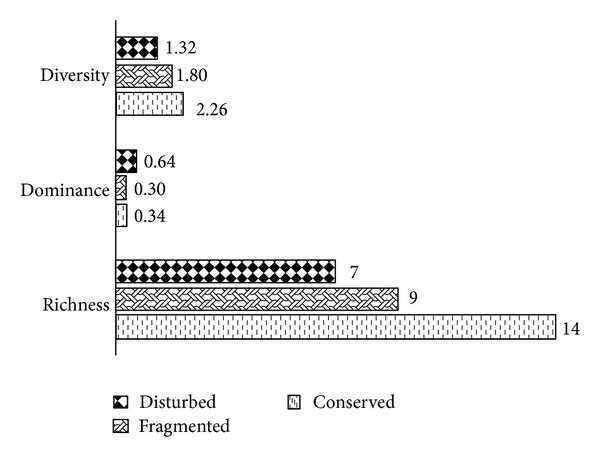
Dominance, diversity, and species richness in the three agroecosystems.

The highest values of diversity and species richness occurred in the most conserved agroecosystem, the forest (2.26 and 14, resp.). Moreover, the values for diversity and species richness decreased with an increasing degree of ecological disturbance, as observed with the cocoa (1.80 and 9) and orange agroecosystems (1.32 and 7, resp.). The dominance level was 0.34 in the forest and increased with an increasing degree of ecological disturbance, with the highest value corresponding to the orange grove agroecosystem (0.64) (Figure 3).

**Figure 4:**
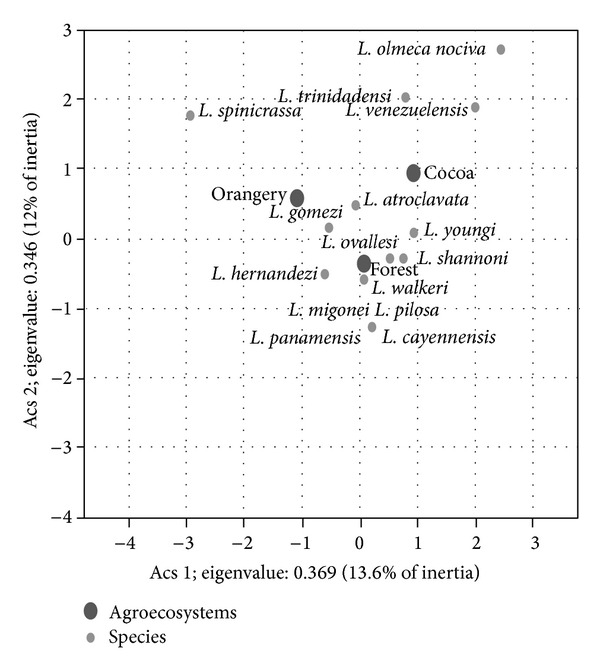
Association between sandflies species and agroecosystems by the simple correspondence analysis.

The simple correspondence analysis between sandflies species and agroecosystems identified a strong association between* L. gomezi* and* L. atroclavata *with disturbed agroecosystems and a strong association between* L. ovallesi, L. walkeri, L. shannoni, L. hernandezi, L. panamensis, L. migonei, L. cayenensis, *and* L. pilosa *with conserved agroecosystems; species such as* L. trinidadensis, L. olmeca nociva, *and* L. spinicrassa *showed no association with any agroecosystem (*χ* ^2^: 124.7; df = 30; *P* = 0.005) (Figure 4). In the conserved agroecosystem,* L. gomezi, L. ovallesi, *and* L. walkeri *demonstrated natural infection with* Leishmania* species, which were identified as the subgenera* Leishmania *and* Viannia*.

## 4. Discussion

Human encroachment on forest ecosystems is driven by logging and agricultural conversion, resulting in sharp and rapidly moving gradients between the relatively cool and humid primary forest and the cultured land, which show strong insolation, higher temperature, and lower humidity. Tropical areas are characterized by a great diversity and wide distribution of sandflies fauna [19, 20]. In Brazil, it has been reported that the devastation of natural areas, which includes natural habitats for sandflies, increases the adaptability of these species to environments with human intervention, as observed by the increasing number of cases of leishmaniasis in urban environments [21].

It is likely that habitat degradation and climate change greatly impact the abundance and richness of sandflies. The results of this study highlight differences in the sandflies population composition and structure across 3 agroecosystems, characterized by the different degrees of ecological disturbance that were surveyed. Few studies on sandflies have focused on this aspect, as most reports have been limited to epidemiological studies and the documentation of naturally infected species [21]. In addition, other studies have focused on how the population composition changes in different areas, such as the home and peridomestic or wild environments [22–24], or according to the type of capture method used [25]. The results are in concert with others who have proposed that changes in habitat may have a marked impact on the sandflies populations [5–10].

The relationship between leishmaniasis and agricultural activity has been recorded and the relationship between coffee cultivation and the transmission of* Leishmania *by sandflies has been recorded in Venezuela, Colombia, Brazil, and Mexico [11, 26–29]. This could be explained by the suitability of shade-grown coffee plantations for the resting and breeding of sandflies. Moreover, this type of agroecosystem presents high biodiversity and promotes the presence of many vertebrates, which in turn act as reservoirs of* Leishmania *and potential feeding sources for sandflies [30, 31].

In this study, the effect of human intervention was reflected in the disturbed agroecosystems as an increase in dominance and a decline in diversity and species richness, relative to less ecologically disturbed areas such as the conserved agroecosystem, where dominance is lower and diversity and species richness are greater. These results are supported by those of previous studies [3, 21, 32]. Most diversity and species richness in the forest, conserved area, could be caused by higher accumulation organic material to accumulate as a result of the decomposition of leaves and vegetation waste lying on the soil favoring larval development.

Environments with significantly disturbed wilderness areas cause certain species to adapt to these new spaces, as observed in our study. Moreover, our results show that anthropogenic modification can favor certain species to colonize these disturbed environments, such as was reported for* L. longipalpis *[33] and* L. flaviscutellata *in urban areas of Brazil [34]. Few species are able to adapt to high levels of anthropogenic disturbance, consequently, demographic parameters such as mortality and birth rates for each species are affected differently, and ecosystem structure and dynamics are in turn affected; yet based on the abundance values, our results suggest that* L. gomezi *was the species with the greatest ability to exploit disturbed environments [3, 35].

Both* L. gomezi *and* L. ovallesi *have been* considered* as important vectors of* Leishmania *[36]. The type of agroecosystem affected the abundance of* L. gomezi *and* L. ovallesi *which have an important effect on the probability of humans being bitten by one of these two vectors.* L. gomezi *has been reported to have a marked preference for biting humans around homes where vegetation is scarce [37], and this species has also been known to invade the inside of the home [36, 37]. These findings suggest a greater risk of transmission of the disease in these areas.

The abundance of* L. ovallesi*, a species that transmits* Leishmania braziliensis, *has also been confirmed as a vector of* Leishmania mexicana *in Venezuela, and in conserved areas such as forests, a potential natural habitat and fragmented areas with cocoa plantations confirm the association of this species with woody vegetation [38–40]. The sympatric relationship between* L. ovallesi* and* L. gomezi* is comparable to what was reported in Brazil between* L. intermedia* and* L. neivai *[21], where* L. ovallesi *is the species with a greater dependence on conserved areas than* L. gomezi*, predominated near the peridomiciles, indicating a process of adaptation, mainly to this environment of less dense vegetation.* L. gomezi *and* L. ovallesi *as predominant species of primary forest, as the deforestation extended, there was a tendency for* L. ovallesi *to disappear, suggesting that this species is more dependent on the primary forest than* L. gomezi*.

In the conserved forest agroecosystem,* L. gomezi, L. ovallesi, *and* L. walkeri *demonstrated natural infection with* Leishmania *of the subgenera* Leishmania *and* Viannia*, and this seems to indicate that these species may be transmitting the leishmaniasis agent in the forest agroecosystem area. If these areas have a greater diversity of sandflies species, it would be expected that there would be a greater coexistence of various species of* Leishmania*, given the specificity between the sandflies vector and* Leishmania*. Moreover, the increased abundance of* L. gomezi* in disturbed agroecosystems indicates that this species has adapted to new environments modified by humans. The altered environments favor adaptation of* L. gomezi*; these results suggest that the transmission pattern may be changing.

This study provides a basis for further in-depth studies to assess how anthropogenic changes can modulate vector composition and distribution and could also help to explain how this might affect the transmission of tegumentary leishmaniasis in Merida and potentially disease risks.

## 5. Conclusion

These results clearly show that sandflies fauna exhibited changes in species number as well as population structure in degraded environments. As a result, changes in the determinants of transmission can lead to the development of new outbreaks.

## Figures and Tables

**Table 1:** Ecoepidemiological characteristics and the degree of disturbance of the agroecosystems.

	Forest	Cocoa	Orangery
Environmental influence			
Temperature	24.8–27.9°C	26.7–32°C	24.8–26.9°C
Relative wetness	65.5–82%	52–75.7%	75.2–83%
Water bodies	Yes	No	No
Type of vegetation	Arboreal	Shrubby and herbaceous	Shrubby
Anthropogenic influence			
Animal presence	No	Yes (domestic)	Yes (domestic and breeding)
Crop presence	No	Yes (cocoa, banana)	Yes (citrus fruit)
Human influence	Felling of trees	Garbage	Chemical contamination
Stored and irrigation water	No	Cistern	Irrigation

Ecological disturbance	Conserved	Fragmented	Disturbed

Level of disturbance	Low	Medium	High

**Table 2:** The species abundance in the agroecosystems studied.

Species	Agroecosystems											
	Forest				Cocoa				Orangery			
	*N*	%	pi	(*λ*)	*N*	%	pi	(*λ*)	*N*	%	pi	(*λ*)
*L*.gomezi*	128	41.16	0.41	0.17	25	29.76	0.30	0.09	73	79.35	0.79	0.63
*L*.ovallesi*	126	40.51	0.41	0.16	36	42.86	0.43	0.18	7	7.61	0.08	0.01
*L*.walkeri*	24	7.72	0.08	0.01	3	3.57	0.04	0.00	3	3.26	0.03	0.00
*L*.trinidadensi**	2	0.64	0.01	0.00	12	14.29	0.14	0.02	5	5.43	0.05	0.00
*L*.panamensis*	11	3.54	0.04	0.00	—	—	—	—	—	—	—	—
*L*.atroclavata**	2	0.64	0.01	0.00	1	1.19	0.01	0.00	1	1.09	0.01	0.00
*L. cayennensis *	1	0.32	0.00	0.00	—	—	—	—	0	—	—	—
*L. hernandezi *	6	1.93	0.02	0.00	—	—	—	—	2	2.17	0.02	0.00
*L*.migonei*	2	0.64	0.01	0.00	—	—	—	—	—	—	—	—
*L. olmeca nociva *	—	—	—	—	1	1.19	0.01	0.00	—	—	—	—
*L. pilosa *	1	0.32	0.00	0.00	—	—	0.00	—	—	—	—	—
*L. puntigeniculata *	2	0.64	0.01	0.00	—	—	0.00	—	—	—	—	—
*L. shannoni *	3	0.96	0.01	0.00	1	1.19	0.01	0.00	—	—	—	—
*L*.spinicrassa*	—	—	—	—	—	—	—	—	1	1.09	0.01	0.00
*L*.venezuelensis**	1	0.32	0.00	0.00	4	4.76	0.05	0.00	—	—	—	—
*L*.youngi*	2	0.64	0.01	0.00	1	1.19	0.01	0.00	—	—	—	—

Total	311	100	1	0.34	84	100	1	0.30	92	100	1	0.64

Number of sandflies (*N*); abundance (pi); Simpson index (*λ*); anthropophilic species (∗); zoophilic species (∗∗).

**Table 3:** Analysis of variance (ANOVA) between the agroecosystems.

Agroecosystems	*α*
Forest-cocoa	0.041*
Forest-orangery	0.073
Cocoa-orangery	0.001*

*Significant differences in multiple comparison (alpha: 0.05).
